# Detection of distant metastases and distant second primary cancers in head and neck squamous cell carcinoma: comparison of [^18^F]FDG PET/MRI and [^18^F]FDG PET/CT

**DOI:** 10.1186/s13244-022-01261-0

**Published:** 2022-07-28

**Authors:** Eirini Katirtzidou, Olivier Rager, Arthur Damien Varoquaux, Antoine Poncet, Vincent Lenoir, Nicolas Dulguerov, Alexandra Platon, Valentina Garibotto, Habib Zaidi, Minerva Becker

**Affiliations:** 1 Division of Radiology, Diagnostic Department, Geneva University Hospitals, University of Geneva, Rue Gabrielle Perret Gentil 4, CH-1205 Geneva, Switzerland; 2 Division of Nuclear Medicine, Diagnostic Department, Geneva University Hospitals, University of Geneva, Geneva, Switzerland; 3 Department of Medical Imaging, Center for Clinical Research Conception University Hospital, University Aix-Marseille, Marseille, France; 4 Center for Magnetic Resonance in Biology and Medicine, La Timone University Hospital, University La Timone, Marseille, France; 5 Center for Clinical Research, Geneva University Hospitals, University of Geneva, Geneva, Switzerland; 6 Clinic for Otorhinolaryngology, Department of Clinical Neurosciences, Geneva University Hospitals, University of Geneva, Geneva, Switzerland

**Keywords:** PET/MRI, PET/CT, Head and neck tumors, Metastases, Second primary cancer

## Abstract

**Purpose:**

This prospective study aimed to compare the diagnostic performance of [^18^]FDG PET/MRI and PET/CT for the detection of distant metastases and distant second primary cancers in patients with head and neck squamous cell carcinoma (HNSCC).

**Methods:**

A total of 103 [^18^F]FDG PET/MRI examinations immediately followed by PET/CT were obtained in 82 consecutive patients for staging of primary HNSCC (*n* = 38), suspected loco-regional recurrence/follow-up (*n* = 41) or unknown primary HNSCC (*n* = 3). Histology and follow-up > 2 years formed the standard of reference. Blinded readers evaluated the anonymized PET/MRI and PET/CT examinations separately using a 5-point Likert score. Statistical analysis included: receiver operating characteristic (ROC) analysis, jackknife alternative free-response ROC (JAFROC) and region-of-interest (ROI)-based ROC to account for data clustering and sensitivity/specificity/accuracy comparisons for a score ≥ 3.

**Results:**

Distant metastases and distant second primary cancers were present in 23/103 (22%) examinations in 16/82 (19.5%) patients, and they were more common in the post-treatment group (11/41, 27%) than in the primary HNSCC group (3/38, 8%), *p* = 0.039. The area under the curve (AUC) per patient/examination/lesion was 0.947 [0.927–1]/0.965 [0.917–1]/0.957 [0.928–0.987] for PET/MRI and 0.975 [0.950–1]/0.968 [0.920–1]/0.944 [0.910–0.979] for PET/CT, respectively (*p* > 0.05). The diagnostic performance of PET/MRI and PET/CT was similar according to JAFROC (*p* = 0.919) and ROI-based ROC analysis (*p* = 0.574). Sensitivity/specificity/accuracy for PET/MRI and PET/CT for a score ≥ 3 was 94%/88%/89% and 94%/91%/91% per patient, 96%/90%/91% and 96%/93%/93% per examination and 95%/85%/90% and 90%/86%/88% per lesion, respectively, *p* > 0.05.

**Conclusions:**

In HNSCC patients, PET/MRI and PET/CT had a high and similar diagnostic performance for detecting distant metastases and distant second primary cancers.

## Key points

- HNSCC patients imaged for suspected loco-regional recurrence/follow-up have more often distant malignant lesions (27%) than patients imaged for primary tumor staging (8%), *p* = 0.03.
- [^18^]FDG PET/MRI has an excellent and similar diagnostic performance as [^18^]FDG PET/CT for detecting distant metastases and distant second primary cancers in HNSCC patients.
- [^18^F]FDG-negative lung nodules with a maximum diameter < 8 mm should be considered as benign in the context of HNSCC.

## Background

Squamous cell carcinomas (SCCs) constitute approximately 95% of all head and neck (HN) cancers [1]. Traditional staging with the TNM system and tailored treatment planning require endoscopic biopsy, imaging (MRI, CT or [^18^F]FDG PET/CT) and information relevant to human papilloma virus (HPV)-positive disease [2]. The detection of distant metastases and second primary cancers in patients with HNSCC is crucial, as it defines treatment strategy and survival. Tumor location and tumor stage affect the incidence of distant metastases [3], which has been reported to be as high as 25% in HNSCC patients, most metastases occurring in the lungs and bones [3–7]. HNSCCs are also associated with a high rate of synchronous or metachronous second primary cancers, most second primary cancers occurring in the HN area followed by the chest [8, 9]. Given the high diagnostic performance of PET/CT to detect malignant lesions, several authors have recommended its routine use for the initial work-up of HNSCC and for imaging at 3–4 months after radio(chemo)therapy [6, 7, 9–11].

Clinical PET/MRI systems have been introduced 10 years ago and in many academic centers they are routinely used in the clinical setting as they allow the acquisition of MRI and PET data in the same patient during a single examination, thus facilitating high-quality fusion of MRI and PET images [12–21]. The advantages of PET/MRI versus PET/CT are mainly the higher soft tissue contrast resolution, more detailed anatomic information and lower radiation exposure. Moreover, the MRI component of PET/MRI systems can perform as a standalone system offering the possibility of functional imaging with diffusion-weighted imaging (DWI) and dynamic contrast-enhanced MRI (DCE-MRI), therefore, making the combination with PET even more promising [13–16, 18]. Some authors have reported the superiority of PET/MRI compared to PET/CT for detecting distant metastases in breast cancer, prostate cancer, gastrointestinal cancer and melanoma, thus allowing an efficient loco-regional and distant staging in one step [15–17]. However, most studies evaluating the diagnostic performance of PET/MRI in HNSCC have focused on assessing primary tumors, lymph node metastases or post-treatment local recurrence [14, 18]. As cross-sectional HN imaging protocols routinely cover the anatomic area between the skull base and the thoracic inlet, second primary cancers located within this region are detected more frequently than second primary cancers located elsewhere.

To the best of our knowledge and as pointed out recently, data on the M staging and on the detection of distant second primary cancers (outside the HN area) by [^18^F]FDG PET/MRI in HNSCC patients are very scarce [18, 19]. Because CT can detect smaller lung nodules than MRI, one may expect a lower diagnostic performance with PET/MRI than PET/CT for lung lesions < 10 mm [22]. This assumption has been challenged by some authors, who reported a superior M staging accuracy with PET/MRI than PET/CT in 27 patients with different HN tumor types [20]. Furthermore, it has been suggested that for cancers originating outside the HN area, the number of missed malignant lung nodules by PET/MRI compared to PET/CT may be negligible [17].

Given these conflicting results and scarce literature, the purpose of this prospective study was to assess and compare the diagnostic performance of [^18^F]FDG PET/MRI and PET/CT for the detection of distant metastases and distant second primary tumors in patients with HNSCC.

## Materials and methods

### Study design, patient inclusion and standard of reference

This prospective study was approved by the institutional ethics committee and was performed according to the guidelines of the Helsinki II Declaration. Inclusion criteria were as follows: (1) adult HNSCC patients who underwent whole-body [^18^F]FDG PET/MRI immediately followed by PET/CT, (2) adequate image quality for image interpretation, (3) appropriate standard of reference to validate findings (i.e., histology and/or follow-up > 2 years after PET/MRI and PET/CT). Exclusion criteria were standard MRI contraindications and refusal to participate. All patients potentially eligible for the study gave their written informed consent, and all patients sustained both examinations without complications. During a period of three years, 88 consecutive HNSCC patients, in whom MRI of the HN and whole-body [^18^F]FDG PET/CT were clinically indicated, underwent a whole-body PET/MRI scan followed by whole-body PET/CT. Both examinations covered the area from the skull base to the mid-thighs. Six patients were excluded from the study because of insufficient image quality (*n* = 2) or because they were lost to follow-up (*n* = 4). Accordingly, 82 patients (70 men, 12 women, mean age ± SD = 61.0 ± 12.0 years) were included. To avoid selection bias, all PET/MRI and PET/CT examinations performed during the study period were included in the analysis. Therefore, during the three-year study period, one patient was examined 7 times, one patient 5 times, 13 patients 2 times, and the remaining 67 patients were examined once with PET/MRI followed by PET/CT, which resulted in a total of 103 PET/MRI and 103 PET/CT examinations in 82 patients. The indication for PET/MRI was staging of histologically proven primary HNSCC (*n* = 38 patients), clinically suspected loco-regional recurrence/follow-up (*n* = 41 patients) and search for the unknown primary HNSCC (*n* = 3 patients). The delay between radiochemotherapy and imaging in post-treatment patients was 16.5 ± 11.3 months.

Histological proof obtained within 2 weeks of PET/MRI and PET/CT was the standard of reference for metastases and second primary cancers in 24 patients, whereas imaging and clinical follow-up > 2 years were the proof of diagnosis in the remaining 58 patients. For lesions without histology after PET/MRI and PET/CT (58 patients), classification into benign versus malignant lesions was done based on the presence versus absence of progression on radiologic examinations obtained during the follow-up period or until death. Criteria for progression were an increase in lesion size (as measured in the axial and coronal planes) during follow-up or the appearance of new lesions. A > 2-year follow-up period was chosen as distant metastases/second primary cancers may be subclinical at initial imaging, and—depending on tumor kinetics and patient immune status, they may show only minimal changes over time [23].

### Imaging protocol

After fasting for 6 h, all patients had a single [^18^F]FDG injection, followed by a PET/MRI and a subsequent PET/CT scan. A mean dose of 373 ± 28 MBq [^18^F]FDG was injected intravenously to all patients before the PET/MRI examination was started. During the time necessary for radiotracer uptake, a dedicated MRI of the HN region (T2-weighted, diffusion-weighted and T1-weighted sequences before and after iv. administration of a gadolinium-based contrast material), followed by total body MRI sequences was performed. The total body MRI sequences (see below) were used for attenuation correction, lesion detection, anatomic localization and lesion characterization. Afterward, a whole-body PET was acquired on the PET/MRI system. The patient was then transferred to the PET/CT scanner, and no additional [^18^F]FDG was injected.

### PET/MRI acquisition

[^18^F]FDG PET/MR imaging was performed on a Philips Ingenuity time-of-flight (TF) PET/MRI (Philips Healthcare, Cleveland, Ohio, USA). A SENSE neurovascular coil was used for the dedicated HN MRI. A whole-body T1-weighted attenuation correction (AC) MRI sequence, referred to as atMR, and a whole-body Dixon sequence using a quadrature body coil followed. These sequences were used for lesion detection and characterization on MRI and for PET/MRI fusion. The parameters for the Dixon sequence obtained after iv. injection of gadoterate meglumine (0.1 mmol /kg Dotarem, Guerbet, Aulnay-sous-Bois, France) were: TE_1_ = 1.1 ms, TE_2_ = 2.0 ms, TR = 3.2 ms, flip angle = 10°, transverse FOV = 450 × 354 mm^2^, voxel size = 0.85 × 0.85 × 3 mm^3^, acquisition time = 2 min 17 s. The 3-tissue-class (lung, soft tissue, air) MRI-based AC procedure described in the literature was applied [24]. The atMR sequence (acquisition time = 2 min 29 s) had the following parameters: TE = 2.3 ms, TR = 4.1 ms, flip angle = 10°, smallest water–fat shift, transverse FOV = 600 mm, slab thickness = 120 mm, voxel size = 1.9 × 1.9 × 6 mm^3^. No additional whole-body T2-weighted or diffusion-weighted sequences were acquired. After obtaining all MRI sequences, PET acquisition was started on average 65 ± 3 min after [^18^F]FDG injection with a total acquisition time = 32 min covering 10 bed positions.

### PET/CT acquisition

PET/CT images were acquired on a Biograph 64 True Point scanner (Siemens Healthcare, Erlangen, Germany). The standard dose CT scan used for attenuation correction and diagnosis (with soft tissue, bone and lung windows) had the following parameters: 120kVp, 180mAs, pitch = 1.2, collimation = 24 × 1.5, 1 s per rotation, reconstructed CT slice thickness = 2 mm, reconstruction interval = 1.5 mm. PET data acquisition was started 120 ± 21 min after injection of [^18^F]FDG with a total of 8–9 bed positions. The delay between the end of the PET acquisition on the PET/MRI machine and the start of the PET acquisition on the PET/CT machine was on average 26 min. No iodine-based iv. contrast material was administered because: (1) according to the ESUR guidelines, for patients with a normal or moderately reduced GFR, there should be ≥ 4 h between injections of gadolinium- and iodine-based contrast agents [25]; (2) current guidelines for PET/CT do not recommend the routine use of iv. iodine-based contrast in all PET/CT examinations [26].

### Image reconstruction

For attenuation correction on the PET/MRI system, the MRI map generated by 3-class segmentation of the atMR sequence was used. A 3-D line-of-response (LOR)–time-of-flight– blob-based OSEM algorithm with standard parameters (3 iterations, 33 subsets, voxel size = 4 × 4 × 4 mm^3^) was employed. On the PET/CT system, the AWOSEM iterative reconstruction algorithm (4 iterations, 8 subsets, voxel size = 4 × 4 × 5 mm^3^) was applied for PET reconstruction. Standardized uptake values (SUVs) were calculated for PET/MRI and PET/CT separately according to the standard formula.

### Image evaluation and SUV measurements

PET/CT and PET/MRI images were evaluated by experienced (> 10 years) board-certified radiologists and nuclear medicine physicians in consensus, each physician contributing their own expertise to the joint reading. Readers were blinded to clinical data and results of other imaging modalities; however, they were aware of the study purpose. PET/MRI and PET/CT examinations were evaluated separately. To avoid recall bias, the interval between PET/MRI and PET/CT readings was ≥ 3 months.

Images were evaluated on a PACS workstation with the Osirix MD Version 11 (http://www.osirixviewer.com) software. The dedicated SUV measurement tool of this software was equally used for region-of-interest (ROI) placement covering the voxels in a lesion volume. For PET/MRI, all areas of focal uptake (uptake > background activity) were first identified on axial PET images after which the atMR and water only Dixon sequence served for image fusion. In analogy, for PET/CT, focal uptake was first identified on PET images, after which CT was employed for anatomic correlation.

For image interpretation, distinction between [^18^F]FDG-positive lesions (uptake > background activity) and [^18^F]FDG-negative lesions was made. In addition, irrespective of focal uptake, morphologic criteria for lesion characterization on MRI/CT were equally applied according to the literature [27, 28]. Therefore, [^18^F]FDG-positive lesions were considered as malignant unless they corresponded to physiologic uptake (e.g., muscle, brown fatty tissue) or unless morphologic criteria suggested a benign etiology, e.g., in the presence of benign fractures, osteoarthritic changes, calcified lymph nodes, lymph nodes with fatty hilar metaplasia, in the presence of non-specific lung infiltrates suggesting an inflammatory origin or in the presence of morphologic characteristics suggesting benign or probably benign lung nodules [27–29]. In addition, in the absence of focal [^18^F]FDG uptake, lung nodules were classified as benign if they were < 8 mm in size.

Lesion size was measured in the axial plane (longest diameter and perpendicular shortest diameter) and in the cranio-caudal direction. Mean and maximum SUV measurements on PET/MRI and PET/CT were carried out for all lesions located outside the HN area. Readers graded lesions using a five-point Likert scale as follows: 1, definitively benign ([^18^F]FDG-negative lesion and clearly benign morphology); 2, probably benign ([^18^F]FDG-negative lesion and probably benign morphology); 3, indeterminate ([^18^F]FDG-positive lesion and non-specific morphology); 4, probably malignant ([^18^F]FDG-positive lesion and probably malignant morphology); and 5, definitively malignant ([^18^F]FDG-positive lesion and clearly malignant morphology). Based on histology and follow-up > 2 years, an independent reader then categorized the lesions as true positive, true negative, false positive and false negative.

### Statistical analysis

Statistical analysis was carried out by an experienced biomedical statistician (> 15 years of experience). Descriptive statistics included the following: number of lesions, lesion size described by mean and standard deviation (SD) and median and interquartile range (IQR) of SUV values measured on PET/MRI and PET/CT. SUV values were log transformed, and the relative differences between SUVs from malignant and benign lesions on the one hand and between SUVs from PET/MRI and those from PET/CT on the other hand were calculated using linear mixed effect models with random effect at two levels: the effect for patients and the effect for examinations within each patient to account for clustering due to multiple examinations per patient and multiple lesions per examination within each patient [30–32]. First, a classic receiver operating characteristic (ROC) approach was used. Because ROC methods have shortcomings when dealing with multiple disease sites, the jackknife alternative free-response receiver operating characteristic (JAFROC) method was also applied [30–32] as the JAFROC method rewards correct lesion localization, while it penalizes incorrect localizations [30]. In addition, a region-of-interest (ROI)-based ROC method, where each image set is partitioned into a constant number of ROIs, was equally employed. Areas under the curve (AUC) were estimated with the JAFROC software (devchakraborty.com), the trapezoidal method under Stata 13 (stata.com) for the inferred-ROC and the Obuchowski method under Stata13 (stata.com) for the ROI-based ROC. The 95% confidence interval [CI] of the AUC for inferred-ROC was estimated according to Delong & Delong [33]; for JAFROC it was based on reader x case ANOVA, and for ROI-based ROC, it was estimated using a bias corrected method. Differences between AUCs between PET/MRI and PET/CT were assessed with the DBM ANOVA algorithm for MRMC ROC data [34]. A two-sided 0.05 level for all analyses was used for statistical significance.

## Results

### Patient and tumor characteristics based on the standard of reference

In the patient group imaged for histologically proven primary HNSCC (*n* = 38 patients, 38 PET/MRI and PET/CT), based on the standard of reference, there were 40 tumors (hypopharynx: *n* = 5; larynx: *n* = 14; oral cavity: *n* = 10; and oropharynx: *n* = 11). Their TNM stage was as follows: T1 (*n* = 2), T2 (*n* = 8), T3 (*n* = 13), T4 (*n* = 17), N0 (*n* = 10), N1 (*n* = 8), N2 (*n* = 22), M0 (*n* = 38) and M1 (*n* = 2). Four patients had stage II disease, 5 patients had stage III disease, and 29 patients had stage IV disease. One patient with stage IV disease had an additional synchronous second primary lung cancer.

In the HNSCC group imaged for suspected loco-regional recurrence/follow-up after radio(chemo)therapy (*n* = 41 patients, 62 PET/MRI and PET/CT), there were 31 histologically proven loco-regional recurrences (T and N) and 5 nodal recurrences (N only). The 31 recurrent tumors (hypopharynx: *n* = 4, larynx: *n* = 8, oral cavity: *n* = 11, oropharynx: *n* = 6; paranasal sinuses: *n* = 2) were classified as follows: rT1 (*n* = 2), rT2 (*n* = 4), rT3 (*n* = 6), rT4 (*n* = 19), rN0 (*n* = 19), rN1 (*n* = 5), rN2 (*n* = 6), rN3 (*n* = 1), rM0 (*n* = 30) and rM1 (*n* = 2). Three out of 5 patients with isolated nodal recurrences also had distant metastases. Five metachronous primary lung tumors were found in 5 patients with local HNSCC recurrence (2 stage III disease and 3 stage IV disease), and one metachronous primary lung tumor was found in a patient without loco-regional recurrence.

In the group with neck node metastases from an unknown primary HNSCC (*n* = 3 patients, 3 PET/MRI and PET/CT), a second primary hepatocellular carcinoma was found in one patient and, in another patient, a second primary lung cancer.

Altogether, distant malignant lesions were present in 23/103 (22.3%) examinations in 16/82 (19.5%) patients. Distant metastases were seen in 14/103 (13.6%) examinations in 7/82 (8.5%) patients, and distant second primary cancers were present in 9/103 (9%) examinations in 9/82 (11%) patients. Patients imaged for suspected neck recurrence/follow-up had more often distant malignant lesions (11/41, 27%) than patients imaged for primary HNSCC (3/38, 8%), *p* = 0.039 (Fisher exact test).

### PET/MRI and PET/CT characteristics of distant metastases and synchronous cancers

Among the 103 examinations included in the study, 23/103 (22%) had distant malignant lesions and 80/103 (78%) did not. A total of 183 distant lesions were identified, among which 82 lesions were malignant, and 101 lesions were benign. Among the 82 distant malignant lesions, 73 were metastases, one lesion was a synchronous second primary lung cancer and 8 lesions were metachronous second primary cancers (7 lung cancers and one hepatocellular carcinoma). Of the 82 malignant lesions, one lesion had no focal [^18^F]FDG uptake on PET/MRI and 6 lesions had no focal [^18^F]FDG uptake on PET/CT; all other malignant lesions showed focal [^18^F]FDG uptake. Among the 35 benign lesions with focal uptake, 32 were detected on PET/MRI and 32 on PET/CT. Sixty-nine lesions showed no focal uptake on both imaging modalities. Benign lesions included lung nodules < 8 mm with no focal uptake, infectious/inflammatory lung lesions and reactive lymph nodes, non-specific pleural thickening and benign bone lesions (benign fractures and degenerative changes), as well as cysts.

Table 1 shows the size and the anatomic distribution of all distant malignant lesions, most malignant lesions being found in the bones (36/82, 43%), lung (24/82, 29%) and mediastinum/hila (17/82, 21%). The mean number of malignant lesions per positive examination (PET/MRI and PET/CT) was 3.6 [range 1–7].

**Table 1:** Size and location of distant malignant lesions (73 metastases and 9 s primary cancers)

	Lesion size in mm (mean diameters ± SD)			
	n	Maximum axial diameter	Minimum axial diameter	Cranio-caudal diameter
Distant malignant lesions	82	17.2 ± 8.5	13.1 ± 6.2	17.3 ± 9.6
Localization				
Lung	24	15.9 ± 12.1	10.7 ± 5.9	14.7 ± 9.8
Mediastinum and hila	17	17.9 ± 4.0	12.5 ± 5.3	20.4 ± 9.5
Bone (spine, ribs, pelvis)	36	17.2 ± 6.8	14.1 ± 6.1	17.6 ± 9.8
Liver	5	23.0 ± 8.4	20.5 ± 7.0	17.2 ± 2.2

Second primary cancers were located in the lung/hila (*n* = 8) and in the liver (*n* = 1)

Table 2 shows the SUVs for all distant lesions (*n* = 117) with focal [^18^F]FDG uptake and *p* values of statistical comparisons between benign and malignant lesions. For both PET/MRI and PET/CT, SUVs were significantly higher in malignant versus benign lesions (*p* < 0.05).

**Table 2:** SUVmean and SUVmax values of distant lesions with focal [^18^F]FDG uptake as measured on PET/MRI and PET/CT and *p* values of statistical comparisons between benign and malignant lesions

	Total number of lesions with focal uptake *n* = 117	Total number of benign lesions with focal uptake *n* = 35	Total number of malignant lesions with focal uptake *n* = 82	*p* value
Number of focal uptake lesions detected on PET/MRI	113	32	81	
SUVmean PET/MRI	2.1 [1.3–3.3]	1.6 [0.9–2.5]	2.3 [1.4–3.6]	0.006
SUVmax PET/MRI	3.1 [1.8–5.1]	2.1 [1.5–3.4]	3.4 [1.9–5.8]	0.003
Number of focal uptake lesions detected on PET/CT	108	32	76	
SUVmean PET/CT	2.2 [1.5–3.2]	2.0 [0.8–2.7]	2.3 [1.6–3.9]	0.003
SUVmax PET/CT	3.4 [2.1–5.1]	2.8 [1.3–3.6]	3.7 [2.1–7.2]	< 0.001

Median values and interquartile intervals are presented. Among the 117 lesions with focal uptake, 104 were detected by the two readers on both modalities, 9 only on PET/MRI and 4 only on PET/CT. Four focal uptake lesions (3 benign and 1 malignant) were missed on PET/MRI, and 9 focal uptake lesions (3 benign and 6 malignant) were missed on PET/CT

There was no statistically significant difference between SUVmean/SUVmax values measured on PET/MRI versus PET/CT neither for distant malignant lesions (*p* = 0.746/* p* = 0.595) nor for distant benign lesions (*p* = 0.373/* p* = 0.476).

Spearman’s rank correlation coefficient showed a statistically significant and strong correlation between SUVmean measurements on PET/MRI and PET/CT for all lesions, Rho = 0.797 [0.682–0.872], *p* < 0.001. Likewise, a statistically significant and strong correlation between SUVmax measurements on PET/MRI and PET/CT was found, Rho = 0.770 [0.634–0.863], *p* < 0.001.

### Diagnostic performance of PET/MRI and PET/CT

In the per lesion analysis, 5/82 malignant lesions were rated as indeterminate (score = 3) on PET/MRI and PET/CT. Four of 82 (5%) malignant lesions were scored as benign (scores 1 and 2) on PET/MRI, and 8/82 (10%) malignant lesions were scored as benign on PET/CT. Therefore, in the per lesion analysis, for a rating ≥ 3, there were only 4 false-negative evaluations with PET/MRI and 8 false-negative evaluations with PET/CT, respectively. Among the 24 malignant lung nodules, 2 nodules in the same patient were rated as benign on both PET/MRI and PET/CT. Among the 17 malignant mediastinal and hilar lesions, all but two were detected by both modalities. Among the 36 malignant bone lesions, all lesions were detected by PET/MRI, while PET/CT missed 4 lesions in the same patient. All malignant liver lesions were detected by both modalities.

In the per examination analysis, only 1/23 (4%) PET/MRI and 1/23 (4%) PET/CT examinations with malignant lesions were rated with a score of 1 (benign); therefore, there was only one false-negative assessment with both techniques. Among the true-negative examinations (*n* = 80), 8 PET/MRI and 6 PET/CT examinations were rated with a score of 3 (indeterminate). Therefore, for a score ≥ 3, there were 8 false-positive assessments with PET/MRI and 6 false-positive assessments with PET/CT. False-positive assessments were caused by non-specific inflammation or sarcoidosis.

**Fig. 1:**
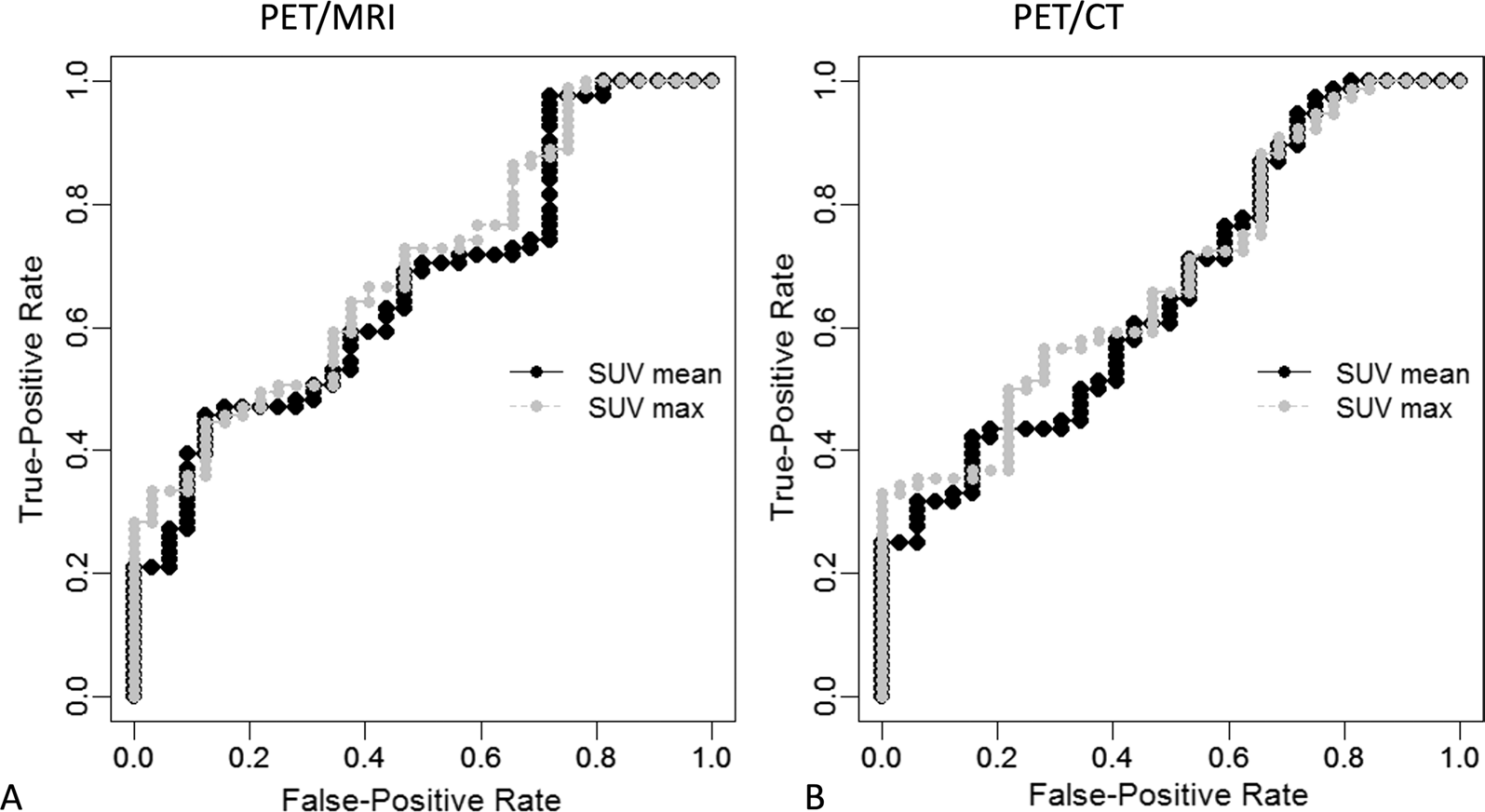
Lesion-based ROC curve analysis of SUVmean and SUVmax values measured on PET/MRI and PET/CT for the detection of distant metastases and distant synchronous cancers. The area under the curve (AUC) for SUVmean and SUVmax measured on PET/MRI was 0.669 [0.562–0.776] and 0.698 [0.596–0.800], respectively. The AUC for SUVmean and SUVmax measured on PET/CT was 0.661 [0.550–0.772] and 0.682 [0.576–0.788], respectively. Delong’s test for 2 correlated ROC curves revealed that SUVmean and SUVmax values measured on PET/MRI and PET/CT did not differ significantly in terms of diagnostic capability (*p* > 0.05)

The lesion-based ROC curve analysis of SUVmean and SUVmax values measured on PET/MRI and PET/CT for the detection of distant metastases and distant synchronous cancers is shown in Fig. 1. There was neither a statistically significant difference between the AUCs of SUVmean values measured on PET/MRI versus PET/CT (*p* = 0.190) nor between the AUCs of SUVmax values measured on PET/MRI versus PET/CT (*p* = 0.138), respectively.

**Fig. 2:**
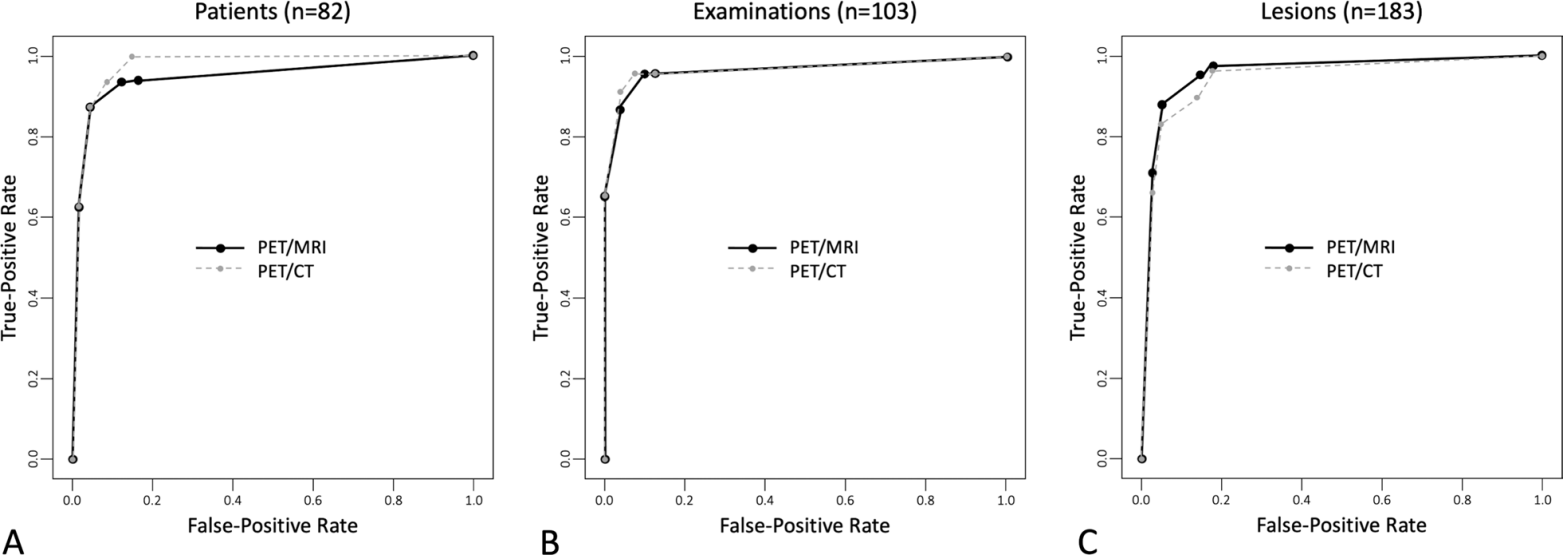
Diagnostic performance of PET/MRI and PET/CT to detect distant metastases and distant synchronous cancers. **A** The AUC for the per patient analysis (*n* = 82) was 0.947 [0.927–1] for PET/MRI and 0.975 [0.950–1] for PET/CT, respectively. There was no statistically significant difference between the two AUCs (*p* = 0.847). **B** The AUC for the per examination analysis (*n* = 103) was 0.965 [0.917–1] for PET/MRI and 0.968 [0.920–1] for PET/CT, respectively. There was no statistically significant difference between the two AUCs (*p* = 0.488). **C** The AUC for the per lesion analysis (*n* = 183) was 0.957 [0.928–0.987] for PET/MRI and 0.944 [0.910–0.979] for PET/CT, respectively. There was no statistically significant difference between the two AUCs (*p* = 0.574)

The diagnostic performance of PET/MRI and PET/CT to detect distant metastases and distant synchronous cancers using the ROC analysis per patient, per examination and per lesion is shown in Fig. 2. There was no statistically significant difference between the AUC of PET/MRI and PET/CT in the ROC analysis per patient (*n* = 82, *p* = 0.847), per examination (*n* = 103, *p* = 0.488) and per lesion (*n* = 183, *p* = 0.574).

The diagnostic performance of PET/MRI and PET/CT to detect distant metastases and distant synchronous cancers using JAFROC analysis was similar for both techniques. The AUC of the JAFROC curve was 0.98 [0.95–1] for PET/MRI and 0.98 [0.95–1] for PET/CT, *p* = 0.919. For the ROI-based ROC analysis (*n* = 8 ROIs per examination, total 824 ROIs), the AUC was 0.98 [0.93–1.00] for PET/MRI and 1.00 [1.00–1.00] for PET/CT, and there was no statistically significant difference between the AUC of the two techniques (*p* = 0.574).

Table 3 shows the diagnostic performance of PET/MRI and PET/CT for a rating ≥ 3. For all pairwise comparisons between PET/MRI and PET/CT, no significant difference was detected between the two techniques. In the per examination/per lesion analysis, the PET/MRI accuracy for distant malignant lesions was 91%/90% and the corresponding PET/CT accuracy was 93%/88%. However, neither PET/MRI nor PET/CT could distinguish between distant metastasis (M1) and distant second primary cancers (T) in 7 cases; therefore, 7 examinations were incorrectly classified as M1 with both imaging techniques. Nevertheless, both PET/MRI and PET/CT correctly indicated the most appropriate target site to perform biopsy thus facilitating further work-up.

**Table 3:** Diagnostic performance of PET/MRI and PET/CT for a score ≥ 3 calculated per patient, per examination and per lesion. 95%CI were calculated using the Clopper–Pearson method

	Analysis per patient (*n* = 82)		Analysis per examination (*n* = 103)		Analysis per lesion (*n* = 183)	
	PET/MRI	PET/CT	PET/MRI	PET/CT	PET/MRI	PET/CT
Sensitivity (%)	94 [70–100]	94 [70–100]	96 [78–100]	96 [78–100]	95 [88–99]	90 [81–95]
Specificity (%)	88 [77–95]	91 [81–97]	90 [81–96]	93 [84–97]	85 [77–91]	86 [78–92]
Accuracy (%)	89 [80–95]	91 [83–96]	91 [84–96]	93 [86–97]	90 [84–94]	88 [82–92]
PPV (%)	65 [49–78]	71 [53–84]	73 [54–88]	79 [59–92]	84 [75–91]	83 [73–90]
NPV (%)	98 [90–100]	98 [90–100]	99 [93–100]	99 [93–100]	96 [89–99]	92 [84–96]
Positive likelihood ratio	7.73 [3.99–14.99]	10.31 [4.76–22.35]	9.57 [4.93–18.57]	12.75 [5.88–27.67]	6.40 [4.00–10.24]	6.46 [3.95–10.57]
Negative likelihood ratio	0.07 [0.01–0.48]	0.07 [0.01–0.46]	0.05 [0.01–0.33]	0.05 [0.01–0.32]	0.06 [0.02–0.15]	0.12 [0.06–0.23]

The McNemar test was used for comparisons (sensitivity, specificity, accuracy). There were no statistically significant differences between PET/MRI and PET/CT (*p* values varied between 0.134 and 1)

PPV, positive predictive value; NPV, negative predictive value

**Fig. 3:**
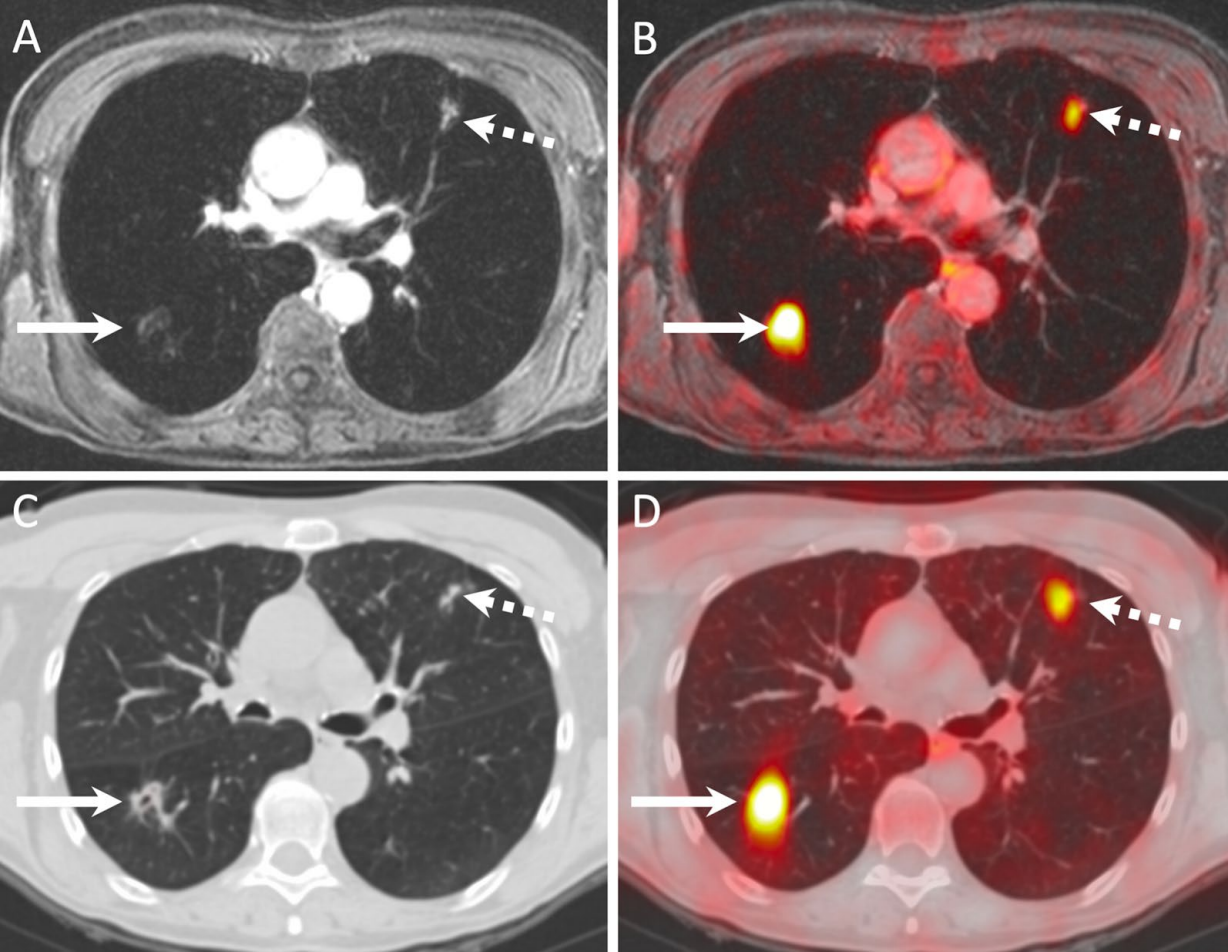
Lung metastases correctly diagnosed on PET/MRI (**A**, **B**) and PET/CT (**C**, **D**) in a 60-year-old female with nodal recurrence after radiochemotherapy for oropharyngeal HPV-negative SCC. The right upper lobe metastasis (arrows) shows a combination of high focal FDG uptake (SUVmax on PET/MRI = 7.7 and SUVmax on PET/CT = 9.3) and an excavated aspect on the contrast-enhanced fat-saturated MR image and on the corresponding CT image. The left upper lobe metastasis (dashed arrows) displays minor focal [^18^F]FDG uptake (SUVmax on PET/MRI = 1.7 and SUVmax on PET/CT = 2.1) and clustered nodules on the corresponding morphologic MRI/CT images. Both lesions were rated with a score of 5 on PET/MRI and PET/CT

**Fig. 4:**
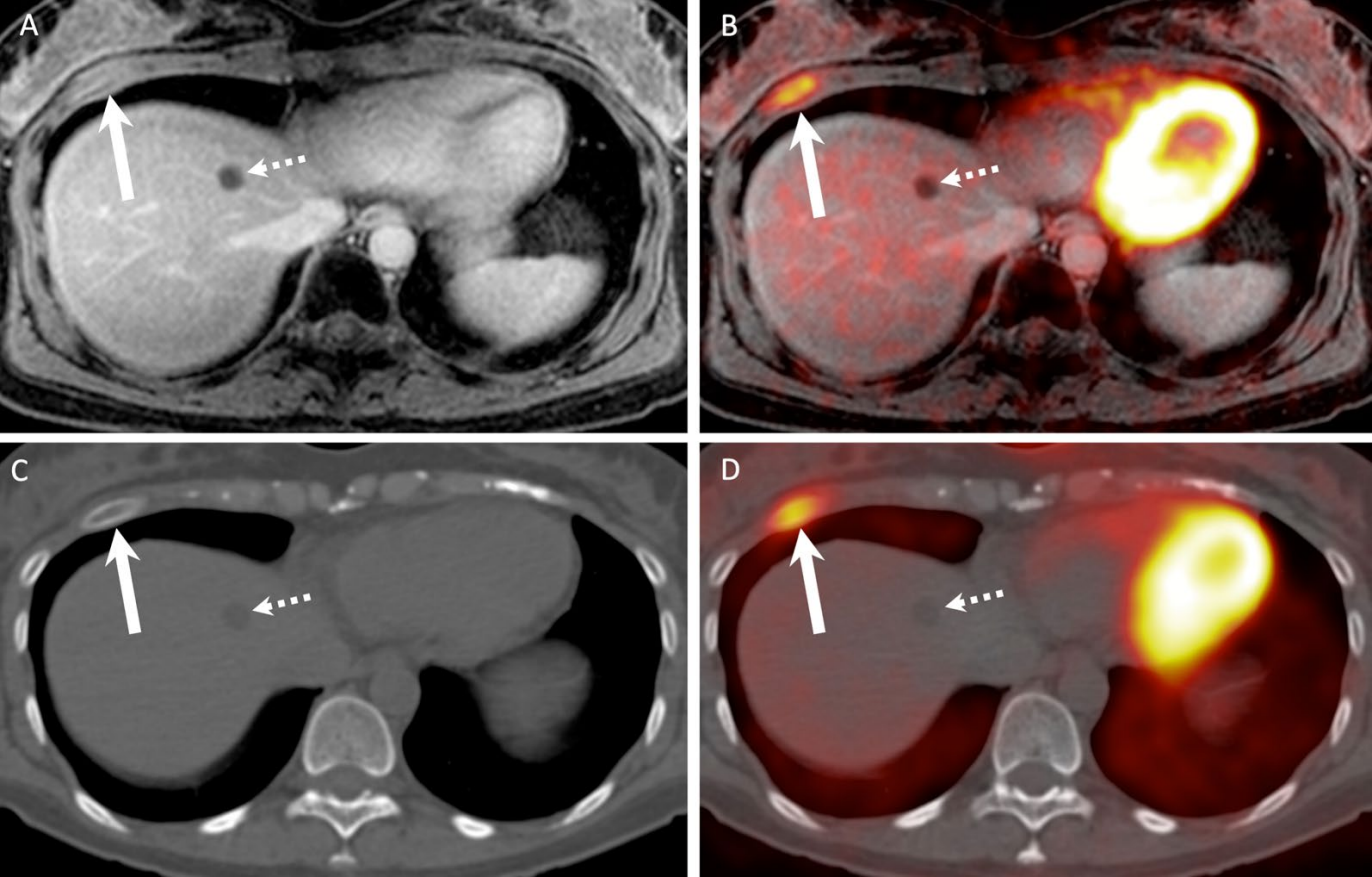
Rib metastasis detected on both PET/MRI (**A**, **B**) and PET/CT (**C**, **D**) in a 42-year-old female without loco-regional recurrence after radiochemotherapy for a SCC of the paranasal sinuses. The rib lesion (arrows) shows a combination of high focal [^18^F]FDG uptake (SUVmax on PET/MRI = 6.3 and SUVmax on PET/CT = 4.7) and an expansile aspect on MRI/CT (arrows). Note lesion enhancement on the contrast-enhanced fat-saturated MR image. The lesion was rated with a score of 5 on PET/MRI and with a score of 4 on PET/CT. Dashed arrows point at a liver cyst

**Fig. 5:**
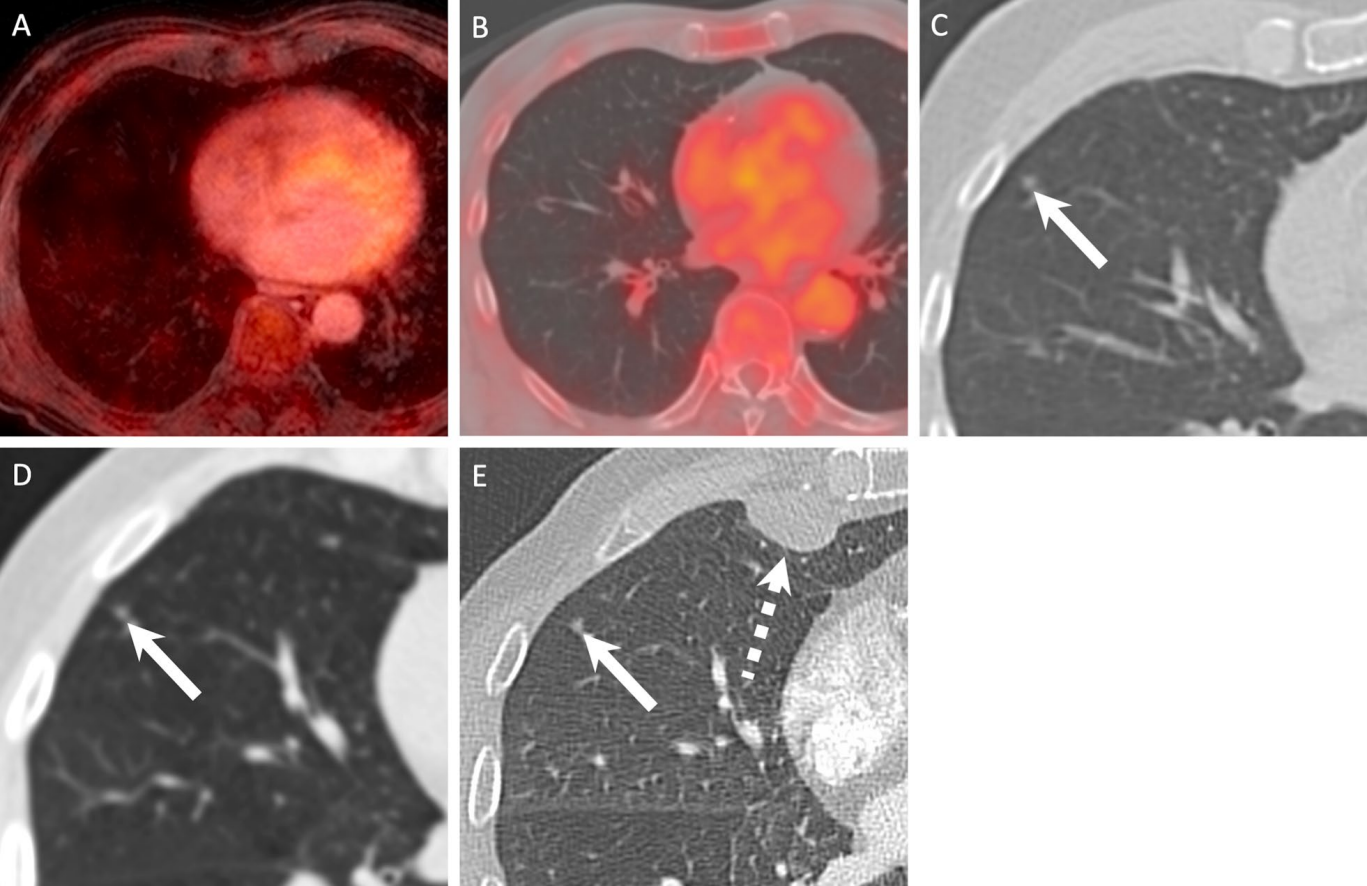
False-negative PET/MRI (**A**) and PET/CT (**B**) in an 89-year-old male with primary SCC of the oral cavity. Both PET/MRI and PET/CT were rated as negative for distant metastases or second primary cancers (diagnostic score = 1). PET/MRI image (**A**) shows no lesion. PET/CT (**B**) and detail of the corresponding CT component of the PET/CT (**C**) show a non-[^18^F]FDG-avid 5 mm solid lung nodule (arrow in **C**), which was considered as benign according to diagnostic criteria (no [^18^F]FDG uptake and < 8 mm in size). The lesion was rated with a score of 1 on PET/MRI and PET/CT. Follow-up CT obtained two months later (**D**) showed no change in size and shape of the 5 mm nodule (arrow). CT obtained 7 months later (**E**) revealed no change in the 5 mm nodule (arrow), however, a pleural metastasis (dashed arrow) that was confirmed histologically. As the pleural metastasis occurred within the 2-year follow-up, both PET/CT and PET/MRI were considered as false negative

**Fig. 6:**
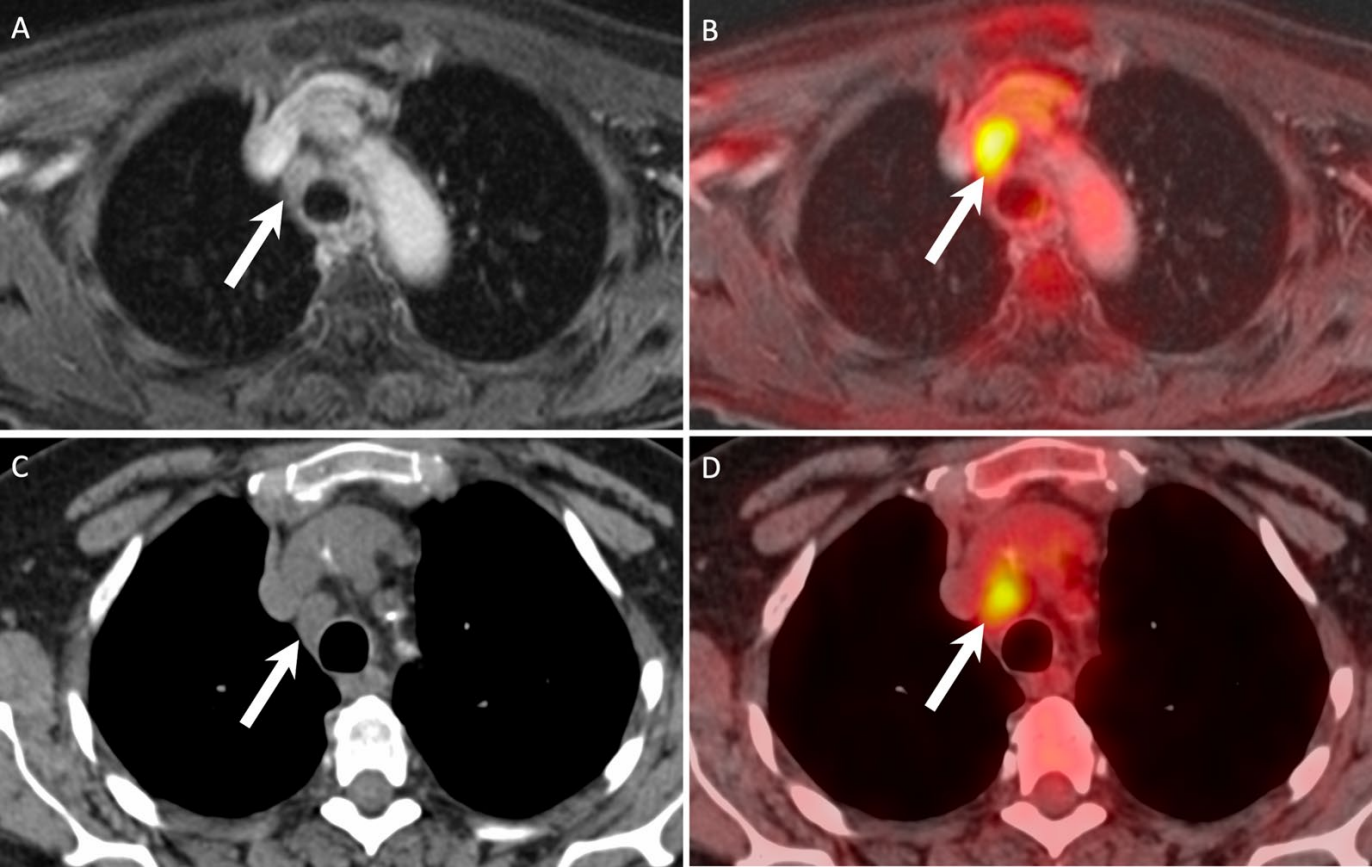
False positive PET/MRI (**A, B**) and PET/CT (**C, D**) in a 63-year-old female imaged for follow-up of a SCC of the larynx (T3N1) treated with radiochemotherapy. Both PET/MRI and PET/CT were rated as positive for distant mediastinal lymph node metastases. An enlarged mediastinal lymph node (arrows) shows a combination of high focal [^18^F]FDG uptake (SUVmax on PET/MRI = 7.5 and SUVmax on PET/CT = 6.7) and slightly heterogeneous contrast enhancement on the contrast-enhanced fat-saturated MR image. On the corresponding CT image, due to the absence of contrast enhancement, only lymph node enlargement was present (13 × 15 × 17 mm). The lymph node was rated with a score of 5 on PET/MRI and a score of 4 on PET/CT. However, mediastinoscopy with biopsy revealed sarcoidosis

Figures 3, 4, 5 and 6 illustrate true-positive, false-positive and false-negative evaluations with PET/MRI and PET/CT.

## Discussion

Detection of distant metastases and second primary cancers in patients with HNSCC is crucial, as it defines treatment choice and prognosis. According to the literature, 4%–24% of patients with primary HNSCCs and up to 26% of patients with recurrent HNSCCs have metastases at presentation, and as many as 1%–22% of all HNSCC patients have second primary cancers [4–7, 19]. Metastases and second primary cancers can occur in the HN imaging area (skull base to thoracic inlet) or at distant sites outside the HN area. In the current study, the prevalence of malignant lesions outside the HN area was 8.5% for metastases and 11% for second primary cancers. In addition, patients imaged for suspected loco-regional recurrence/follow-up had significantly more often distant malignant lesions than patients imaged for primary tumors.

PET/CT and CT are routinely used to detect distant malignant lesions in HNSCC patients; however, the introduction of PET/MRI has challenged the role of PET/CT. In this study, we have compared the diagnostic performance of PET/MRI and PET/CT to detect distant metastases and distant second primary cancers. Our study showed that irrespective of the type of analysis performed (ROC per patient, ROC per examination, ROC per lesion, JAFROC, ROI-based ROC or binary analysis with a score ≥ 3), the two modalities had a similar and high diagnostic performance in HNSCC patients.

Although some authors have recently suggested that PET/MRI could be reliably used for the characterization of pulmonary lesions [35], it is important to have independent confirmation of the validity of this finding in the context of staging and restaging of tumors frequently associated with lung lesions, such as HNSCC. In a series of patients with different types of primary cancers, PET/MRI had a similar sensitivity as PET/CT for [^18^F]FDG-avid pulmonary nodules, but the sensitivity of PET/MRI was significantly lower than the sensitivity of PET/CT for small [^18^F]FDG-negative nodules [36]. This lower sensitivity for small [^18^F]FDG-negative nodules has been attributed to the inferior spatial resolution of morphologic MRI sequences compared to CT [37, 38].

Depending on histology, some cancer types are strongly [^18^F]FDG avid, whereas others are not. In consequence, [^18^F]FDG-avidity of specific tumors influences the diagnostic performance of [^18^F]FDG PET, and studies should, therefore, consider this fact. Based on data obtained in patients with different tumor types (but no HNSCC), some authors have suggested that [^18^F]FDG-negative sub-centimeter lung nodules may be benign in > 98% of patients [18, 36, 38]. One important observation in our patient cohort is the fact that irrespective of the technique used (PET/MRI or PET/CT), it appears that in HNSCC patients, metastases and distant second primary cancers are in the vast majority of cases [^18^F]FDG positive; therefore, their detection rate based on combined morphologic and metabolic evaluation was similar to both modalities, resulting in > 95% sensitivity and a NPV of 99% in the per examination analysis. Our study also showed that [^18^F]FDG-negative lung nodules < 8 mm in the context of HNSCC were benign in most cases.

Some authors have reported that in patients with various malignant tumors (but no HNSCC), PET/MRI could depict more liver lesions than PET/CT and with a higher diagnostic confidence for small lesions [39, 40]. It was equally shown that contrast-enhanced MRI and DWI complement each other in the liver, and adding DWI sequences had a clinical impact in 10% of patients with liver lesions [41]. In our study, there were only 5 liver lesions in 4 patients, among which one lesion was a hepatocellular carcinoma, the other lesions being metastases. Due to the small number of liver lesions in this patient cohort, no conclusive results regarding the diagnostic performance of PET/MRI versus PET/CT for the specific evaluation of liver lesions in HNSCC patients can be drawn.

Regarding bone metastases from different cancer types and primary bone malignancies, some authors reported a similar diagnostic performance of PET/MRI and PET/CT for lesion detection, localization and evaluation of margins. In contrast, other authors using a similar study design as in this series concluded that PET/MRI was superior to PET/CT in certain cancer types, e.g., breast cancer because contrast-enhanced T1-weighted images enabled detection of bone lesions with very faint or absent [^18^F]FDG PET uptake [15, 42]. In the current study, PET/MRI detected all bone metastases from HNSCC, while PET/CT missed 4 lesions due to their poorer conspicuity on PET/CT versus PET/MRI.

Some authors have focused on discrepancies between PET/MRI and PET/CT and have pointed out that PET/MRI may be superior to PET/CT for the overall clinical management of oncologic patients [43]. However, in our series, due to the similar detection rate of distant malignant lesions with both techniques, we could not confirm these observations.

Recently, it has been shown that the diagnostic performance of PET/MRI is similar to that of PET/CT in primary oropharyngeal and hypopharyngeal SCC in terms of detection of metastases and second primary cancers [19]. While the study of Yeh et al. addressed metastases and second primary cancers in the HN region and in distant sites together, our study specifically focused on malignant lesions located outside the HN area. Current practice in most institutions includes whole-body PETCT or PET/MRI at 3–4 months post-treatment, and currently, there are no recommendations regarding imaging of distant anatomic areas in HNSCC patients with suspected loco-regional recurrence or follow-up beyond 3–4 months post-radiotherapy. However, our study suggests that, because of the high prevalence of distant metastases and distant second primary cancers occurring during follow-up (27% in this study), total body imaging with [^18^F]FDG PET/CT or with [^18^F]FDG PET/MRI should be considered more often. Furthermore, as [^18^F]FDG PET/MRI has been shown to provide excellent results for detecting local recurrence after radio(chemo)therapy [14], loco-regional assessment can, therefore, be reliably complemented with whole-body PET/MRI for distant assessment.

Limitations of the current study include the fact that we did not use whole-body diffusion-weighted sequences in the PET/MRI protocol, nor did we use contrast-enhanced CT in the PET/CT protocol, both of which could potentially have contributed to improved lesion detection. Also, newer simultaneous PET/MRI equipment with high-resolution thin slice sequences may have superior results. Nevertheless, regarding PET/MRI, some authors have pointed out that gadolinium-enhanced images (as available in the current study) provide the highest diagnostic accuracy for the detection of distant lesions, and – in the presence of PET data – the diagnostic yield of additional DWI images is limited. Regarding PET/CT, we applied the standard protocol used in most publications, which do not routinely use iv. iodine-based contrast material. Also, the current guidelines for PET/CT do not recommend the routine administration of iv. iodine-based contrast in all PET/CT examinations [26]. Furthermore, based on the ESUR guidelines, for patients with a normal or slightly reduced GFR, a > 4 h interval should be observed if gadolinium-based and iodine-based contrast is administered intravenously the same day [25]. Therefore, the standard dose unenhanced CT (albeit with soft tissue, lung and bone windows) of the PET/CT examination was done without iv. contrast administration. As most metastases and most second primary cancers in HNSCC patients are expected to be in the lung/mediastinum or in the bones, one may also argue whether iv. contrast material is absolutely necessary for the detection of lesions in these locations.

Another potential limitation of the current study is the fact that the order of examinations (PET/MRI followed by PET/CT) was the same for all patients. The rationale behind this approach was that MRI sequences covering the HN and total body were acquired during the time necessary for radiotracer uptake (60 min), thus reducing the total time the patient was required to remain still and minimize moving and speaking. Therefore, the delay between radiotracer injection and PET acquisition was longer for PET/CT as opposed to PET/MRI. Nevertheless, to the best of our knowledge, the vast majority of published studies using a single dose of [^18^F]FDG and same-day PET/MRI and PET/CT have also used the same order of examinations for all patients, either first PET/MRI and then PET/CT [15, 44, 45] or vice versa PET/CT followed by PET/MRI [13, 17, 19, 22]. In these studies, the delay between [^18^F]FDG injection and the second PET examination was longer (90–150 min) than between [^18^F]FDG injection and the first PET examination (60 min), both delays being similar to the delays seen in our study. In addition, some authors reported that delayed acquisitions may have advantages over acquisitions at 1 h post-[^18^F]FDG injection due to a slight increase in tracer uptake in malignant lesions and a modest decrease in uptake in benign lesions, respectively [46, 47].

## Conclusions

In conclusion, in our cohort of patients with HNSCC, both [^18^F]FDG PET/MRI and PET/CT had an excellent and similar diagnostic performance for the detection of distant metastases and distant second primary cancers. In HNSCC patients, distant metastases and distant second primary cancers were in the vast majority of cases [^18^F]FDG positive; therefore, both imaging modalities had a NPV of 98% and 99% in the per patient and per examination analysis, respectively. Our study also showed that [^18^F]FDG-negative lung nodules with a maximum diameter < 8 mm were benign in most HNSCC patients.

## Data Availability

The datasets generated during and/or analyzed during the current study are available from the corresponding author on reasonable request.

## Abbreviations

DCE-MRI: Dynamic contrast-enhanced MRI
DWI: Diffusion-weighted imaging
HNSCC: Head and neck squamous cell carcinoma
JAFROC: Jackknife alternative free-response receiver operating characteristic
ROC: Receiver operating characteristic
ROI: Region-of-interest
